# Exploring the use of nanofluids in pump-free systems for solar thermal applications

**DOI:** 10.1038/s41598-023-44375-1

**Published:** 2023-10-10

**Authors:** Anna Kosinska, Boris V. Balakin, Pawel Kosinski

**Affiliations:** 1 Department of Mechanical and Marine Engineering, Western Norway University of Applied Sciences, Bergen, Norway; 2 Department of Physics and Technology, University of Bergen, Bergen, Norway

**Keywords:** Chemical engineering, Nanoparticles, Fluid dynamics

## Abstract

By using nanofluids as a working fluid in pump-free designs, thermal energy systems can become more efficient and have reduced maintenance costs, ultimately extending the system’s lifespan. In this paper, our goal is to investigate unsteady phenomena in the irradiation process and highlight their significance. To accomplish this, we conducted a series of experiments using a square loop of glass pipes filled with carbon black nanofluids and irradiated with a halogen lamp to simulate solar irradiation. The resulting convective motion of the nanofluids allowed us to observe the performance of different concentrations of carbon black, with 0.005–0.01 wt.% proving to be the most effective. Additionally, we identified unsteady processes that occur at the beginning of the process or when the irradiation changes. Finally, we employed computational fluid dynamics simulations to gain further insight into these phenomena.

## Introduction

Solar energy is a renewable and sustainable source of energy that can be harnessed to generate electricity and heat. The two major types of systems used to take advantage of this energy are solar photovoltaic and thermal systems. Photovoltaic systems convert sunlight into electricity, while thermal systems use the sun’s energy to heat a medium, which, for example, can be a liquid.

A solar collector is an essential component of thermal systems. It is a heat-exchanging device that absorbs incident solar radiation and converts it into thermal energy. Solar collectors in these systems have traditionally used fluids such as water as the absorber. However, to improve the efficiency of these systems, researchers have begun incorporating nanofluids instead of water.

Nanofluids are a type of fluid that is prepared by suspending nanomaterials, for instance, metal oxides, ceramics, and carbon nanostructured materials, into base fluids, such as water or oil. These particles, measuring only a few nanometres in size, have unique properties that enhance the heat transfer and thermal conductivity of the fluid. As a result, the use of nanofluids in solar collectors can increase the efficiency of these systems, enabling them to produce more energy with less input.

Carbon nanomaterials have been found to be particularly promising for preparing nanofluids and heat transfer application due to their thermophysical properties. Research studies revealed that carbon-based nanofluids boost the efficiency of various types of solar collectors, including flat-plate, evacuated-tube, parabolic trough, and hybrid photovoltaic thermal solar collector. Borode et al.^1^ presented a review of papers where the use of carbon-based materials in nanofluids was emphasized. In the following, we give a short description of some recent papers, not mentioned by Borode et al.

Zheng et al.^2^ discussed the use of water-based mono-walled carbon nanotube (MWCNT) and hybrid MWCNT-TiN nanofluids in direct absorption solar collectors (DASCs) to enhance light absorption. They indicated that the MWCNT nanofluids have high solar absorption capabilities, and that the inclusion of TiN nanoparticles further improves absorption performance.

In the paper by Sheikholeslami et al.^3^, numerical simulations were conducted to study the turbulent flow of a carbon nanotube (CNT)-water system within a circular tube solar system. A complex swirl flow device was incorporated into the circular tube to enhance fluid-wall impingement and achieve warmer fluid. The study also examined the impact of mirror concentration, geometric parameters, and different configurations on optical performance and thermal efficiency. Nanofluids with CNT were also investigated numerically by, e.g., Turkyilmazoglu^4^.

Zuo et al.^5^ investigated the potential of using collagen-based carbon black nanofluids for solar thermal applications. They discovered that these nanofluids have good heat transfer performance and light absorption, as well as high photothermal efficiency.

The paper by Zakaria et al.^6^ described experiments where they tested how well different types of nanofluids, specifically those containing carbon nanotubes, improved the absorption efficiency of solar collectors. They conducted the experiment outdoors using both flat-plate and evacuated-tube collectors to produce distilled water.

Singh et al.^7^ discussed the use of graphene oxide nanofluids as a heat transfer fluid in concentrated solar power. They carried out experiments and simulations to evaluate the thermal conductivity and heat capacity enhancement.

The work by Rafiei et al.^8^ researched hybrid energy conversion system that generates power and freshwater using a desalination system, an organic Rankine cycle system, and a solar parabolic trough concentrator. The study examined the use of different nanoparticles, among others MWCNT in oil as the base fluid, and investigated their influence on the performance of the system.

The study of Sani et al.^9^ reports the optical characterization of a new fluid comprising single-wall carbon nanohorns and ethylene glycol for solar energy applications. The nanohorns enhance sunlight absorption compared to the base fluid and outperform conventional carbon forms like carbon-black particles.

Qu et al.^10^ looked into the optical absorption and photo-thermal conversion properties of nanofluids made of graphene oxide and water. They discovered that increasing the amount of graphene oxide or decreasing the size of graphene oxide sheets increases the nanofluids’ optical absorption capacity. The study also found that there are optimal concentrations of graphene oxide for maximum temperature rises in a direct absorption solar collector, and that these optimal concentrations depend on the size of the sheets. Also, Li et al.^11^ explored the use of graphene-based nanofluids in DASCs to enhance solar absorption and photothermal conversion performance. The study found that the addition of a small amount of single-layer graphene or graphene oxide significantly improves the photothermal conversion efficiency of the base fluid, which increases with the concentration.

Pramanik et al.^12^ explored the use of directly solar radiation absorbing nanofluids, which contain amorphous-carbon nanoparticles, in solar thermal collectors. They applied both experiments and simulations. Kuzmenkov et al.^13^ considered the potential use of nanoparticles in solar-driven desalination, specifically in a photothermal boiling. An experimental study was conducted using a laboratory-scale system and three types of nanoparticles (multi-wall carbon nanotubes, iron oxide particles, and a commercial paste based on carbon nanotubes) at various concentrations. Also, in their another paper^14^, these researchers aimed to develop an experiment and theory that describes the photothermal boiling in aqueous suspensions of graphite.

Although nanofluids offer enhanced heat transfer properties for applications like DASCs, they also present challenges such as particle sedimentation, high production costs, corrosion risks, material compatibility issues, potential environmental concerns due to nanoparticle toxicity, system design complexity, lack of standardization, and limited long-term performance data. These disadvantages highlight the need to carefully assess and address these issues before the widespread adoption of nanofluids in various systems, including DASCs.

Fluids in the thermal energy systems are usually circulated by pumps. Therefore, one way to improve the efficiency of the thermal energy systems is by using pump-free designs. This is also the main objective of this paper. Using nanofluids as the working fluid is a viable approach to attain this goal. The high thermal conductivity of the nanomaterials in nanofluids may allow for efficient heat transfer without the need for pumps. This not only increases the efficiency of the system, but also reduces the maintenance costs. Additionally, pump-free systems also have less moving parts, which reduces the chances of system breakdowns and prolongs the life of the system.

Another advantage of using nanofluids in pump-free solar thermal systems is that they have a higher thermal capacity than traditional fluids, which means they can store more heat. This is beneficial for systems that need to provide heat for extended periods, such as in residential or industrial applications. Nanofluids may lead to better performance of the system, resulting in more energy output.

The next objective of this paper is to investigate unsteady phenomena that may occur at the start of the irradiation process or due to sudden changes in the irradiation conditions. Unlike many previous researchers who focused on the long-term steady-state performance, we emphasize the importance of examining these unsteady phenomena, which sets our research apart from the existing literature.

## Experimental set-up

To begin the process, a specific amount of carbon black (CB) (ENSACO 350G from Timcal) was mixed with distilled water, and an equal amount of sodium dodecyl sulfate was added to maintain the stability of the nanofluid. Thus, we followed the strategy used in oue previous research^15–17^. The mixture was stirred using a conventional ceramic magnetic stirrer (VWR VMS-C4 Advanced) for 20 min. Then, the suspension was subjected to treatment in a Branson 3510 (130 W, 40 kHz) ultrasonic bath for an hour. It should be noted that this method has also been used in our previous studies^15–17^. This technique produces homogeneous nanofluids that remain stable for at least several months.

The nanofluid concentrations obtained in this study were 0.001, 0.0025, 0.005, 0.010, 0.020, 0.035, and 0.050 wt.%. These concentrations were lower than those used in our previous works but were more comparable to those used by Struchalin et al.^18^, who used carbon nanotubes. Since we used CB nanoparticles, it is worth comparing the effect of the carbon structure.

**Figure 1:**
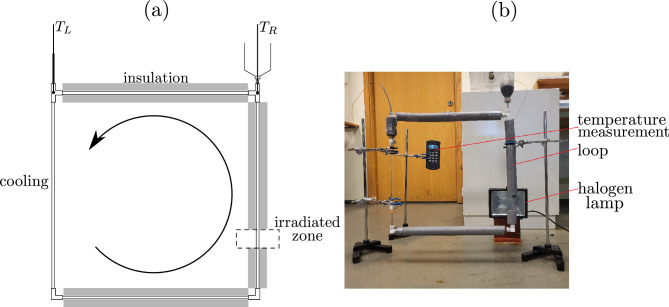
(**a**) Schematic of the experimental set-up (not to scale). $T_L$ and $T_R$ denote locations of the two thermocouples used in the measurements; (**b**) photo of the rig (The protective screen was removed to avoid obscuring the details).

The schematic of the experimental set-up is depicted in Fig. 1. The working fluid was placed in a system of straight pipes forming a square loop. Each of the loop sides had a length of 50 cm.

The pipes were made of glass, with internal and external diameters of 4 mm and 6 mm, respectively. The pipe on the right-hand side of the loop was exposed to irradiation within a zone measuring 3 cm in vertical width/height. This zone was situated at a vertical distance of 38 cm from the topmost pipe and 10 cm from the surface of a halogen lamp (400 W/230 V). The light intensity was 4.38 kW m$^{-2}$ according to our previous measurements^16^. The lamp was positioned perpendicular to the plane of the loop.

All the pipes were insulated except for the pipe on the left-hand side of the loop where the cooling process occurred (natural convection), with the ambient temperature of 18 $^{\circ }$C. The insulation was made of polyethylene foam with the outer diameter of 36 mm.

The upper corners were open to atmosphere, enabling filling the system with the working fluid using the container depicted at the right corner and avoiding air bubble formation. The openings also made it possible to measure the temperature in the corners. For this, we employed K-type thermocouples ($T_L$ and $T_R$). The temperatures were recorded using a Multilogger Thermometer (HH506RA) from Omega Engineering. We emphasize that thermocouple $T_R$ offers more intriguing results due to its proximity to the light source. For each concentration, we conducted five experiments to ensure the repeatability of the results.

In our investigation, we opted not to include a scenario with a pump in the system, as this alteration would result in a complete change of the configuration. It’s worth noting that pump-assisted systems, specifically DASCs, have already been thoroughly explored in previous research.

## Experimental results

**Figure 2:**
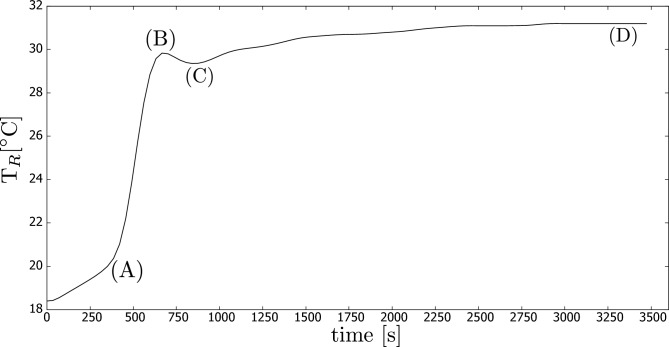
Temperature ($T_R$) history for one experiment, in which carbon black concentration was 0.005 wt.%.

The temperature history recorded by thermocouple $T_R$ is presented in Fig. 2 for the case where the CB concentration was 0.005 wt.%. Similar temperature behaviour was observed for the other cases as well, but they are not included in this paper.

During the experimental process, we observed that the temperature increased slowly initially (up to point A as shown in Fig. 2) due to the heating of the entire set-up. Subsequently, the temperature increased sharply as the heated portion of fluid arrived, reaching its maximum at point B. It is noteworthy that the temperature then decreased (up to point C), which is explained by the fact that the portion of fluid was initially quiescent, allowing it to absorb more heat before moving due to natural convection, as confirmed by the computer simulations presented later in this paper. In the final stage of the process, the temperature of the entire experimental rig continued to increase.

**Figure 3:**
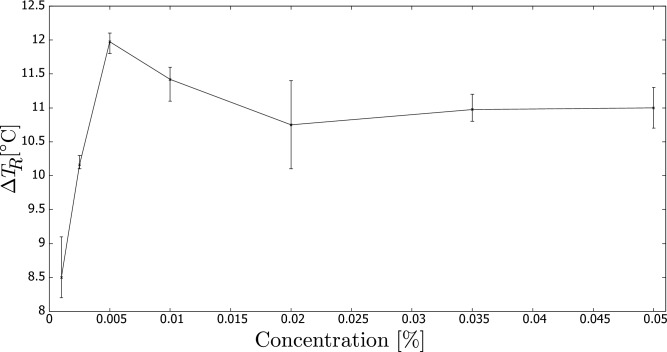
Temperature ($T_R$) increase from initial to the temperature measured in (B) (defined in Fig. 2) vs. carbon black concentration (wt.%).

Next, Fig. 3 presents the results obtained from all the studied concentrations, depicting the maximum temperature ($T_R$) at point (B). It is worth noting that there is a sharp increase in performance observed at the lowest concentration. The optimal concentration range for achieving the maximum temperature lies between 0.005-0.010%. However, there is only a moderate decrease in performance at higher concentrations.

**Figure 4:**
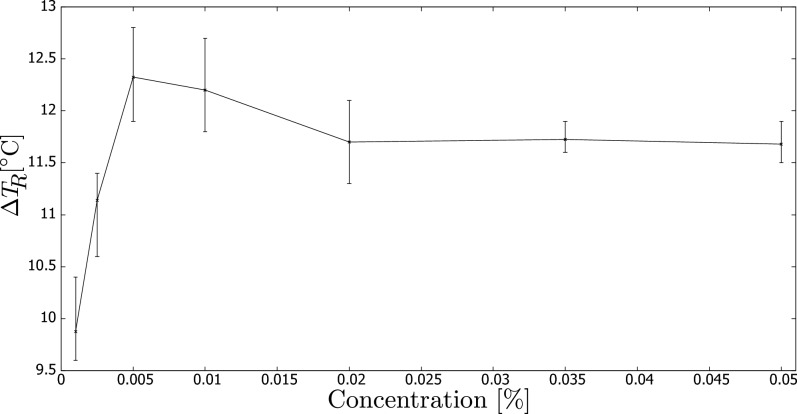
Temperature ($T_R$) increase from the start to the end of temperature measurement vs. carbon black concentration (wt.%).

Similar conclusions can be drawn from Fig. 4, which reports the temperature increase at the end of the measurement using the same sensor.

**Figure 5:**
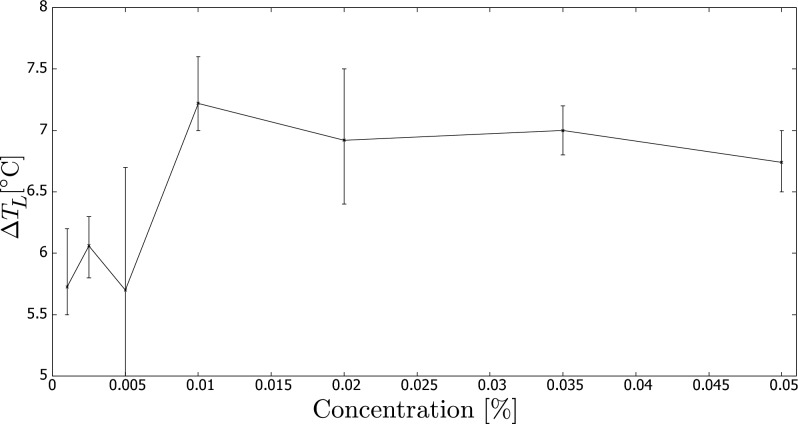
Maximum temperature increase relative to initial temperature in experiment vs. carbon black concentration (wt.%) using sensor $T_L$.

Finally, Fig. 5 displays the maximum temperature rise measured by sensor $T_L$. Similar to the previous analysis using sensor $T_R$, we compare the influence of nanoparticle concentration. However, we notice that the temperature differences are lower than those detected by sensor $T_R$ due to the zone below sensor $T_L$ not being insulated, resulting in heat loss. Nevertheless, we obtain a comparable pattern as discussed earlier, with a relatively rapid increase in performance for the lowest concentrations. On the other hand, the maximum temperature increase occurred at a concentration of 0.01%, rather than 0.005% as observed with sensor $T_R$.

Here, we compare our results with those reported in the literature. For instance, in our previous paper^17^, we investigated the performance of a DASC consisting of a set of pipes irradiated by a halogen lamp. In line with the current study, we used the same type of nanofluid, and the maximum performance of the setup was also observed at concentrations around 0.005–0.010%. In another study^19^, multi-walled carbon nanotubes dispersed in a water-ethanol base were tested in a DASC. However, they also observed the maximum performance at 0.005–0.010%, despite using a different type of nanofluid.

Finally, Zuo et al.^5^ tested the photothermal conversion of CB with bone collagen peptide nanofluid and found that the best efficiency was achieved at a concentration of 0.005% CB, similar to our findings and those of the previous studies.

Hence, it is noteworthy that our simplified experimental setup yielded comparable results to those of previous studies, despite differences in the geometry and working fluids used in DASCs. This indicates that our system could serve as a useful and cost-effective alternative for researchers interested in investigating the photothermal performance of nanofluids.

The obtained results can be theoretically interpreted using the Beer-Lambert law, which expresses the decrease in radiation intensity, *I*, with increasing propagation distance, *x*, within the fluid as follows:

1 $$\begin{aligned} I = I_0 \exp \bigg [ -Kx \bigg ], \end{aligned}$$

where *K* is the extinction coefficient, and the peak radiation intensity, $I_o$, is observed at the surface where *x* = 0.

Next, we introduce an absorption length, *L*, which is the depth at which the intensity becomes $1/e \approx 0.368$ of the peak radiation. Thus, from the above:

2 $$\begin{aligned} L = \frac{1}{K}. \end{aligned}$$

Therefore, we can assume that the maximum performance of the system is achieved when the pipe diameter is less than the absorption length, allowing for light absorption to take place throughout the entire depth of the fluid. In other words:

3 $$\begin{aligned} D < L \rightarrow K_{min} = \frac{1}{D}, \end{aligned}$$

where $K_{min}$ becomes the lowest allowed extinction coefficient. As the pipe diameter was 4 mm, $K_{min} = 250$ m$^{-1}$ in our research.

We must also emphasize that if the extinction coefficient is higher than $K_{min}$ (or for larger pipe diameters), not all of the fluid volume will be subject to irradiation, and the light absorption process will occur primarily close to the irradiated side of the pipe. This situation can result in unnecessary thermal losses to the surroundings from this high-temperature zone.

The extinction coefficient of a nanofluid depends largely on the properties of the base fluid and the concentration of nanoparticles. For example, when using CB nanoparticles in water, we can refer to a model presented in^19^:

4 $$\begin{aligned} K = K_{base} + \frac{3}{2} \alpha _p A, \end{aligned}$$

where: *A* = 2.3$\cdot$10$^6$m$^{-1}$ (an empirical constant), $\alpha _p$ is the nanoparticle concentration and $K_{base}$ is the extinction coefficient of the base fluid.

In our latest research, as described in^17^, we determined that $K_{base}$ was 186 m$^{-1}$ for the same type of halogen lamp (i.e., the wavelength spectrum) and a fluid depth of 5 mm. However, in another paper of ours, as cited in^15^, we estimated $K_{base}$ to be 37 m$^{-1}$ for larger fluid depths. Using Eq. (4) and these two values of the extinction coefficient, we can estimate that the minimum particle concentration varies between 0.002 and 0.006%. Interestingly, this range is consistent with the optimum concentration that we found in our experiments.

Our results can also be compared to those reported in^20^, where a similar set-up was used with a magnetic nanofluid in a loop irradiated by a halogen lamp. However, in that paper, the velocities obtained were not sufficient to drive the flow without the help of an external magnetic field. Nonetheless, our system differed by using carbon-based particles, and our nanoparticle concentrations were significantly lower than those used in the reference study.

## Modelling

We conducted CFD simulations as the next step in our research using the commercial software Star-CCM+, version 13.06.012. To reduce computational time, we simplified the computational domain by excluding some parts of the experimental setup from our model, as explained below.

It is important to emphasize that the irradiation and convective flow in the pipes involve extremely large time scales. Therefore, we chose to focus on the initial phase of the process. Moreover, the pipe geometry was quite challenging to discretize in the CFD software we used. The small diameter of the pipes required a fine mesh, which would result in a vast number of computational cells, especially when considering the much longer pipe lengths.

**Figure 6:**
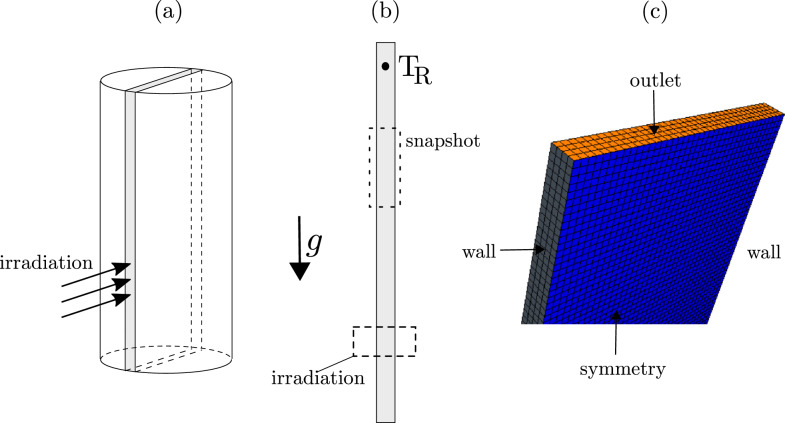
Schematic of the computational domain: (**a**) the circular system is replaced by a ‘flat’ system to reduce computational time; (**b**) the projection of the flat system with the irradiation zone and temperature sensor indicated; (**c**) the computational mesh used in the simulations with boundary conditions. Further, the rectangle ‘snapshot’ (between 23 and 31 cm measured from the bottom) represents a zone within which the temperature and pressure fields will be presented subsequently (in Fig. 8).

As a result, we investigated only the right vertical pipe of the experimental set-up because the irradiation process and the initial upward motion of the heated fluid occur there. In addition, we did not consider the whole pipe cross-section, but rather limited the computational domain to a thin ‘slice’ cut from the pipe, as depicted in Fig. 6a.

The computational domain was 40 cm high, 4 mm wide, and 0.4 mm thick. The irradiation zone was located between 9 cm and 12 cm from the bottom. A monitor point, where the temperature was recorded, was situated 16 cm above the upper edge of the irradiation zone (see Fig. 6b).

In other words, the size of the irradiation zone, the distance between the zone and the monitor point, and the width were identical to those in the experimental setup. The length of the region below the irradiation zone and the thickness of the computational domain were arbitrarily selected after some initial tests.

In our simulations, we used regular cubic computational cells, as illustrated in Fig. 6c. The total number of cells was 480,000, which was determined after conducting a series of simulations to test grid-dependency.

For the irradiated and opposite walls, we employed non-slip boundary conditions denoted as ‘wall’ in Fig. 6c. The bottom and upper boundaries were simulated using pressure outlet boundary conditions^21^, meaning that the values of variables such as velocity on the boundary of the solution domain are extrapolated from within the domain. The two remaining boundaries were modelled as slip wall denoted as ‘symmetry’ in Fig. 6c.

The software solved the governing Navier-Stokes equations for an incompressible flow with thermal properties similar to water. The presence of particles was negligible due to their very low concentrations. Therefore, the main thermal properties were stated to be the same as for water. We also employed the Boussinesq approximation to simulate the buoyancy effect.

To discretize the equations, we used central differences to divide the spatial intervals. The time was advanced using the Euler implicit method with a step of 50 ms. We obtained numerical solutions for the governing equations using the SIMPLE numerical scheme with relaxation coefficients of 0.8 for velocity and 0.2 for pressure^21^.

In addition to the standard Navier-Stokes equations, the software also solved the equation of energy to model the temperature effects. We included a volumetric heat generation in the irradiation zone as a source term in this equation. This was modelled as shown below.

In our analysis, we assumed the radiation to be one-dimensional as shown in Fig. 6a. We selected the value of $I_o$ = 4384 W m$^{-2}$ to correspond to the measured light intensity at the distance between the irradiated pipe and lamp (10 cm). This value was acquired during our previous research, reported in^16^.

The volumetric heat generation, $q_v$, was later calculated by differentiating the above relation:

5 $$\begin{aligned} q_v = - \frac{\textrm{d}I}{\textrm{d}x} = I_0 K \exp \bigg [-Kx \bigg ]. \end{aligned}$$

Based on this, we calculated the overall heat production by calculating the average of the above function:

6 $$\begin{aligned} \overline{q_v}=\frac{1}{d} \int _0^d q_v(x) \textrm{d}x = \frac{I_o }{d}\bigg [1-\exp (-Kd)\bigg ], \end{aligned}$$

where *d* is the width of the computation domain (the pipe internal diameter).

The selection of the extinction coefficient is generally not straightforward in mathematical modelling. In addition to the fluid properties and light wavelength, this coefficient also depends on the fluid depth subject to irradiation, due to the Rayleigh scattering (for more information, see^17^). Therefore, following^17^, we tested these values of the coefficient: 186 m$^{-1}$ for water (corresponding to the lowest nanoparticle concentrations), 730 m$^{-1}$ for the optimum concentration corresponding to 0.005–0.010%, and, for comparison, 5000 m$^{-1}$, which mimics significantly higher concentrations.

In addition, we considered two cases of heat loss to the surroundings. In the first case, no heat loss was accounted for. In the second case, convective heat losses were modelled using the Newton’s law of cooling with the heat transfer coefficient of 10 W (m$^{-2}$ K$^{-1}$), while the radiative heat loss was accounted for by using the emissivity of 1.0. Nevertheless, the observed differences did not influence the conclusions shown in the following section, where the simulation results are presented.

## Simulation results

**Figure 7:**
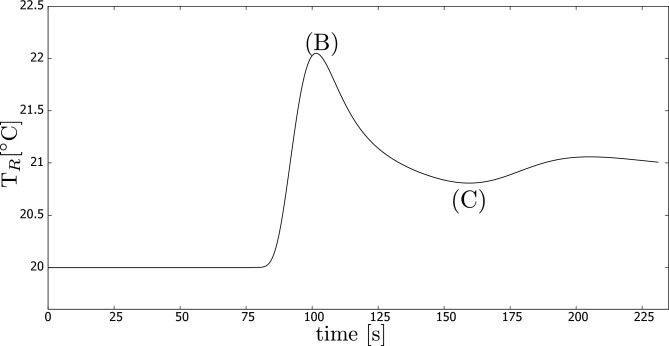
Computer simulations: temperature ($T_R$) history for *K* = 186 m$^{-1}$.

The history of temperature measured at the monitoring point associated with the experimental sensor $T_R$ is presented in Fig. 7. In this simulation, we used the lowest studied extinction coefficient, but the other simulations lead to a similar trend. These results can be directly compared to the experimental results depicted in Fig. 2, which were discussed previously in the paper.

It is worth noticing that the shape of the temperature history from both simulations and experiments is similar. We observe the rapid increase to the maximum temperature (denoted as (B) in Figs. 2 and 7). Finally, the temperature drops to point (C) before it increases further.

**Figure 8:**
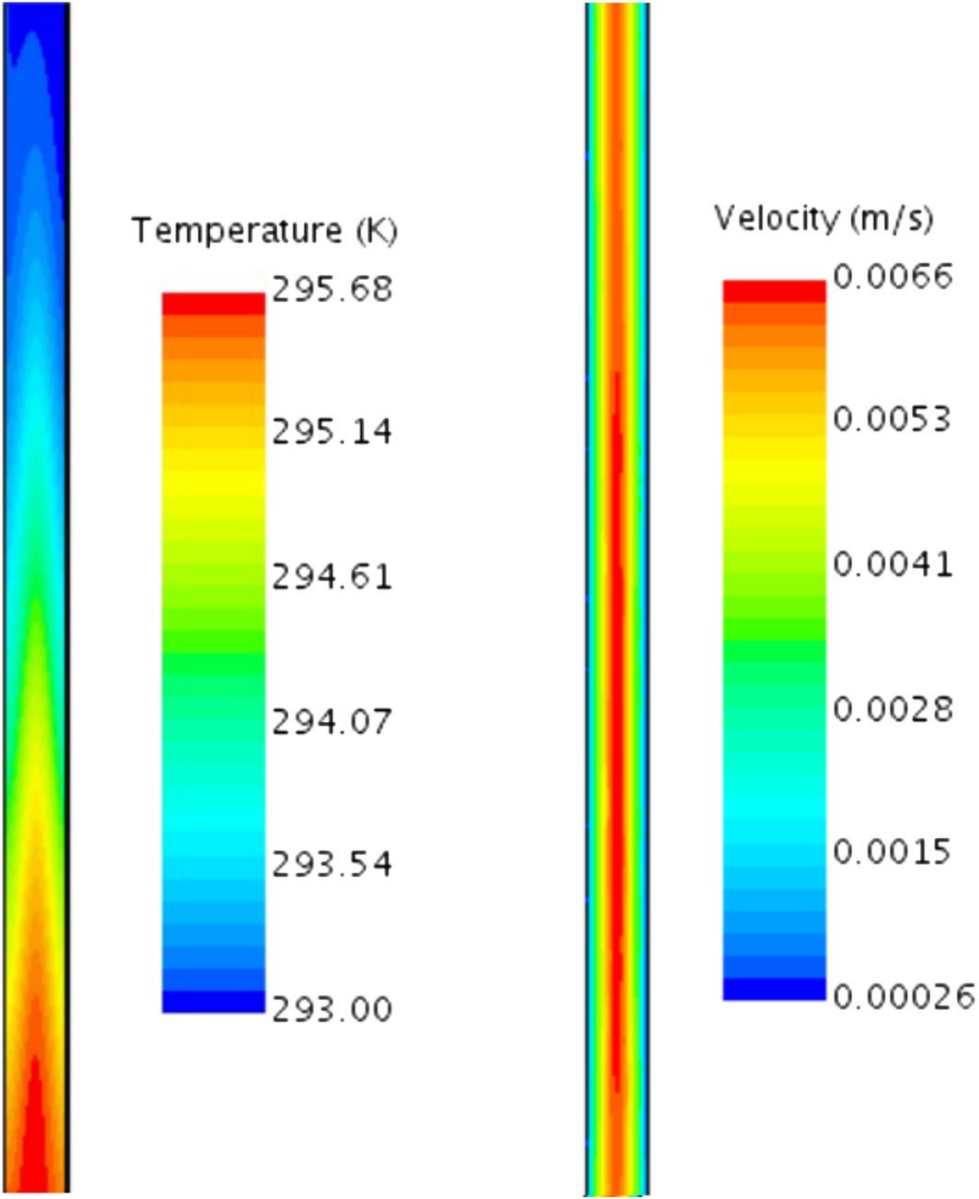
Temperature and velocity magnitude in the geometry zone depicted as ‘snapshot’ in Fig. 6. The extinction coefficient was 186 m$^{-1}$.

The potential explanation given in the previous section is confirmed upon investigating the temperature field as a function of time. A snapshot is presented in Fig. 8 (the left image) for a section of the system corresponding to the area labelled as ’snapshot’ in Fig. 6b.

We observe a ‘wave’ of heated fluid travelling upward through the system. This region was created during the initial stage of the process when the fluid was not yet in motion.

Nevertheless, the initial increase to point (A), detected during the experiments, did not occur in the simulations. As discussed previously, this was a result of the heating of the whole experimental rig by the halogen lamp. This special situation was not modelled in the simulations.

Generally, as also discussed in^17^, the actual temperature increase observed in the experiments is lower than the one obtained from the simulations. This also refers to the time scale of the process. The halogen lamps not only heat the fluid through radiation absorption but also raises the temperature of the glass wall in the pipe system, leading to higher heat transfer to the fluid due to the radiating lamps. As mentioned in the previous paragraph, due to the complexity of this effect, it was not considered in the simulations. Thus, the primary objective of this study was to conduct a qualitative comparison between the investigated fluids to assess their relative performance, following the same approach as initiated in^17^.

Therefore, we showcase the temperature increase of a studied nanofluid, $\Delta T_{NF}$, relative to the temperature increase of the base fluid (water), $\Delta T_w$. The temperature increases of the nanofluids and water are as measured in point (B) in Figs. 2 or 7. From this, we define a dimensionless temperature increase: $\Delta T_{NF}/\Delta T_w$.

**Figure 9:**
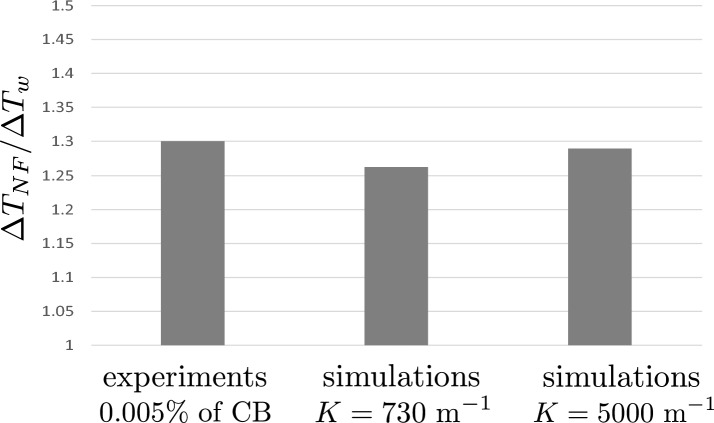
Relative temperature increase (of nanofluids vs. water) for the experiments and simulations.

These findings are displayed in Fig. 9 and focus on three cases: the experiments (for CB concentration of 0.005%), the simulations with *K* = 730 m$^{-1}$, and the simulations with *K* = 5000 m$^{-1}$. Thus, Fig. 9 provides a comparison between the simulations and the experiments, demonstrating a high degree of concurrence between the two.

Figure 8 (the right image) shows a snapshot of velocity magnitude for the same part of the computational domain. Here, it is interesting to investigate the value of this maximum fluid velocity. For the case where *K* was 186 m$^{-1}$, the velocity was 0.0066 m s$^{-1}$, as also depicted in the figure. For the case, were *K* was 730 m$^{-1}$ the velocity was slightly higher and equal to 0.0082 m s$^{-1}$. Finally, if *K* was 5000 m$^{-1}$, the maximum velocity magnitude was 0.0088 m s$^{-1}$.

The difference between the two last cases is not significant, these results also confirm that the increase of particle concentration (or the extinction coefficient) does not really improve the performance. This was also observed in the experiments. It is also worth noting that Balakin et al^20^ obtained a similar order of magnitude of velocities using a similar setup but with a different type of nanofluid, as discussed earlier.

## Concluding remarks

In this research, we utilized a simple experimental set-up to investigate the performance of CB nanofluids for potential application in DASCs. The set-up consisted of a closed loop to facilitate the study of natural convection. Consequently, no pump was employed in the system, enabling us to focus exclusively on the behaviour of the fluid. Additionally, our objective was to examine the unsteady effects, an aspect that had not been extensively explored in existing literature.

It is important to mention that the results obtained in this study aligned with those reported by other researchers. This consistency was also confirmed through theoretical analysis and computer simulations of the process. The extinction coefficient served as the primary parameter in the theoretical component of our research.

The computational domain used in the simulations was deliberately simplified, enabling computationally efficient numerical experiments. Despite the simplifications, the conclusions drawn remained consistent.

Our research primarily focused on the short-term effects occurring in systems involving nanofluids exposed to solar irradiation. These phenomena can be critical not only during system start-ups but also when there are changes in irradiation conditions throughout daylight hours.

## Data Availability

The data is available from the corresponding author upon reasonable request.
